# Supplementary figures and images for: Lineage trajectories and fate determinants of postnatal neural stem cells and ependymal cells in the developing ventricular zone
Source: PLoS Biol. 2025 Jul 30;23(7):e3003318. doi: 10.1371/journal.pbio.3003318 (PMC12327645; doi:10.1371/journal.pbio.3003318)

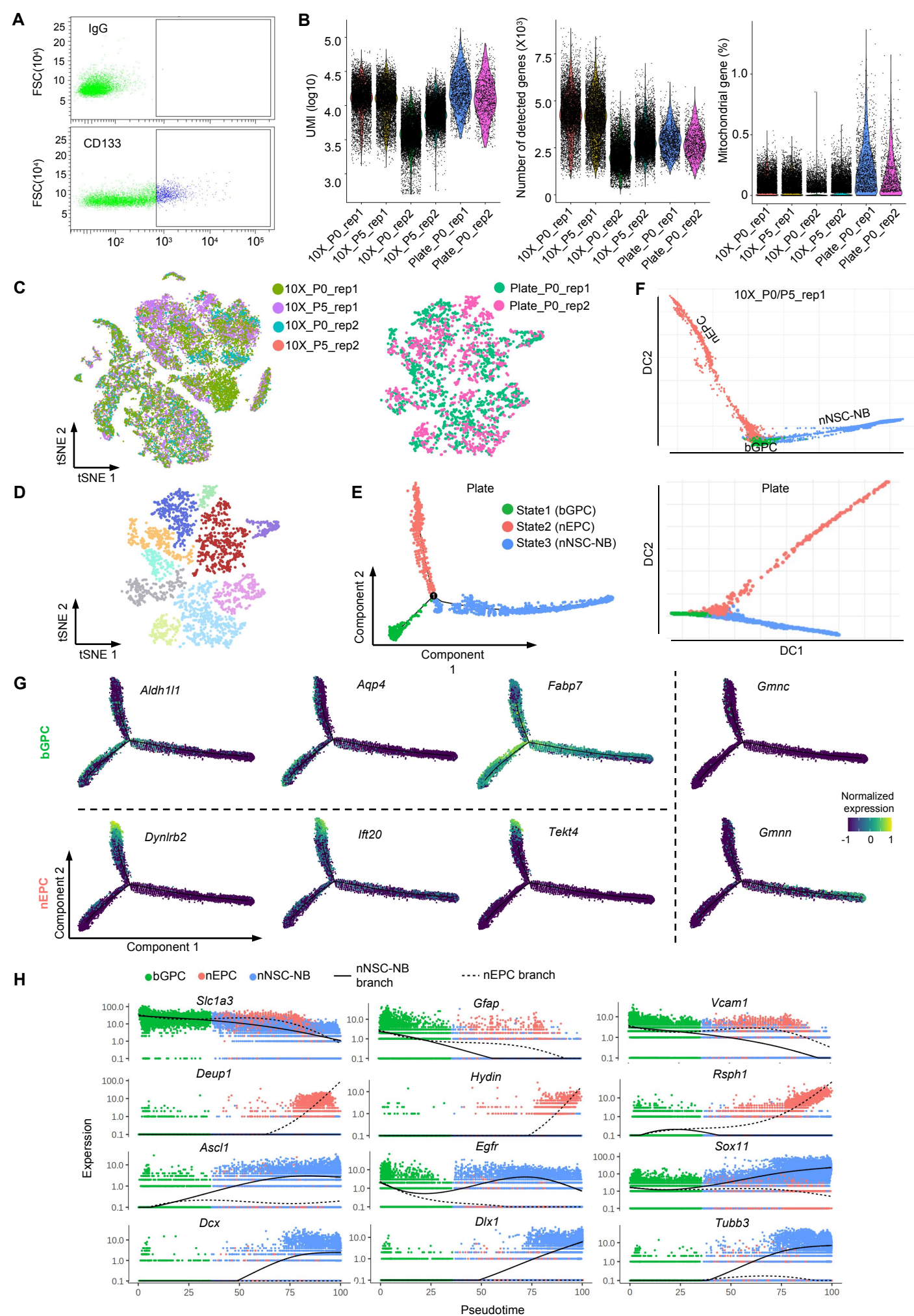

Supplementary Figure 1

Supplement: S1 Fig — (A) Distribution and sorting gates of CD133-labeled cells. IgG served as a negative control for defining the sorting region (indicated by the rectangle in the bottom plot). (B) UMI count, gene count, and mitochondrial gene percentage for each individual cell in each replicate of 10× and the Plate data. (C) 2D t-SNE visualization of 30,445 cells from the 10× data (left) and 2,594 cells from the Plate data (right). Individual cells are color-coded according to replicates. The replicates of 10× data were integrated using Harmony algorithm. (D and E) 2D t-SNE visualization (D) of the Plate data and the bifurcating trajectory (E) constructed by Monocle. Individual cells are color-coded according to clusters or states. (F) The bifurcating trajectory inferred using the diffusion map algorithm. (G) Expression profiles of additional markers used to assign cell classifications of bGPCs, nEPCs and nNSC-NBs. (H) Expression dynamics of various marker genes over pseudotime. (PDF) [file pbio.3003318.s001.pdf]

A

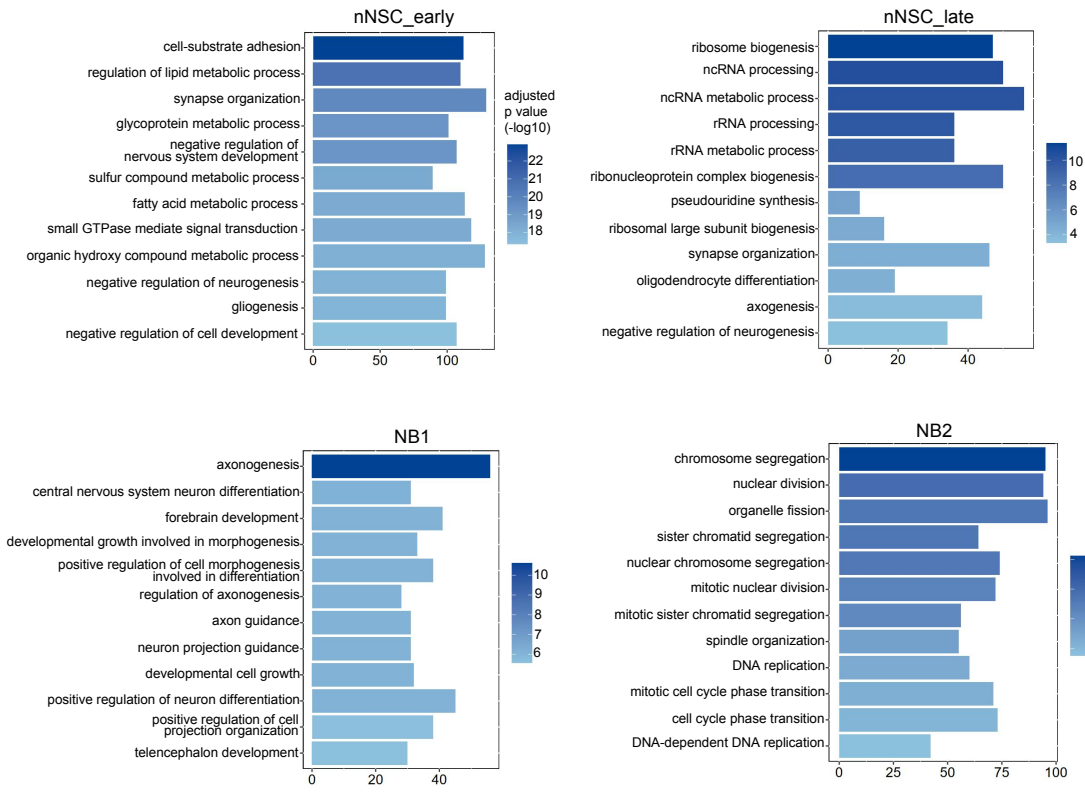

B

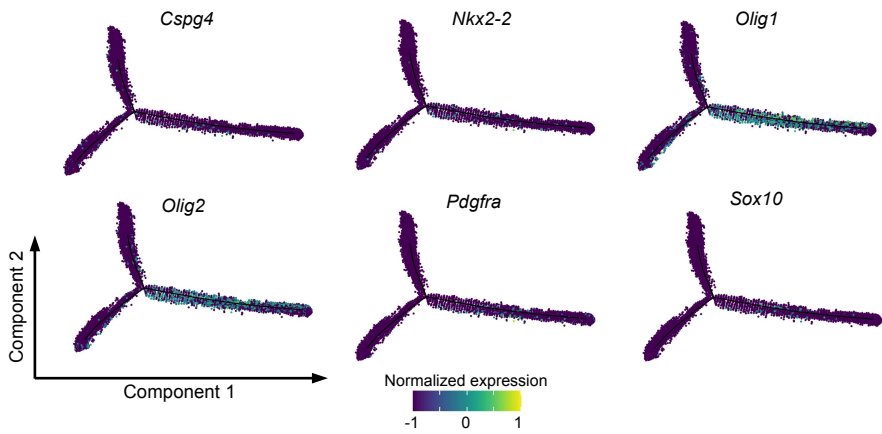

C

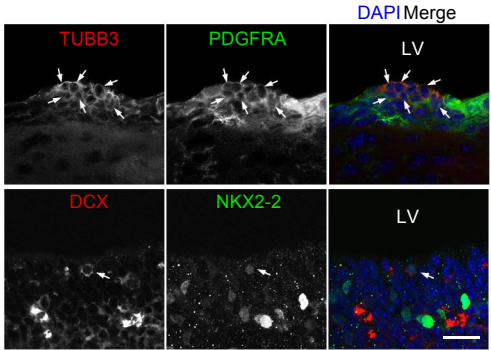

Supplementary Figure 2

Supplement: S2 Fig — (A) Top 12 GO terms of biological processes from genes differentially expressed in the four clusters along the nNSC-NB branch. (B) Expression profiles of six well-known OPC marker genes along the bifurcating trajectory. (C) Immunofluorescence analyses of neonatal brain sections showing the co-expression of the NB marker (TUBB3, DCX) and the OPC marker (PDGFRA or NKX2-2). Arrows indicate double-positive cells. LV, lateral ventricle. The scale bar is 20 μm. (PDF) [file pbio.3003318.s002.pdf]

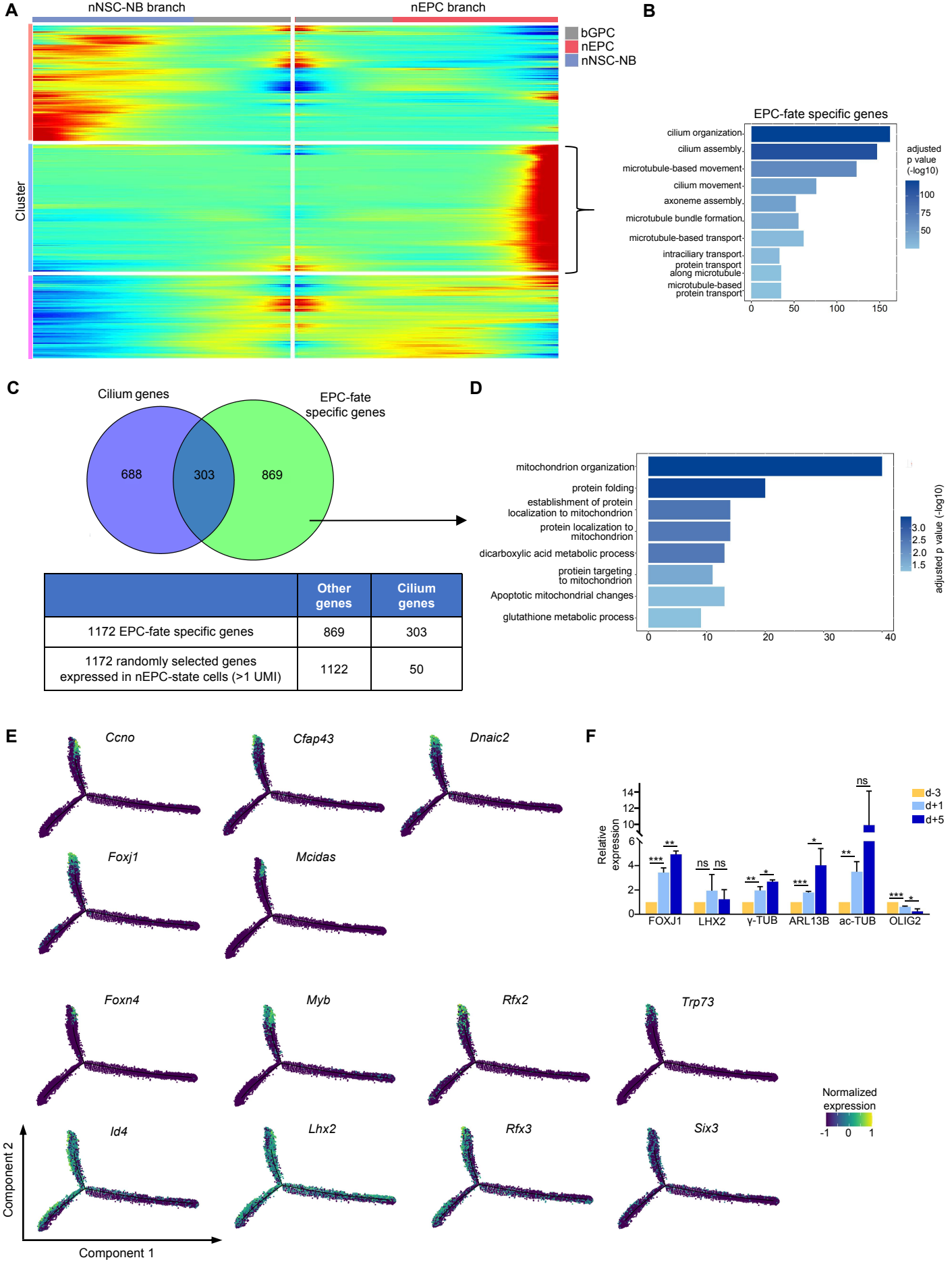

Supplementary Figure 4

Supplement: S4 Fig — (A) Heatmap showing the dynamic expression of the top 3,000 branch-specific differentially expressed genes. (B) Top 10 GO terms of biological processes enriched in 1,172 EPC-fate specific genes. (C) The Venn diagram illustrating that 303 EPC-fate specific genes are included in the cilium gene set and the table showing the high enrichment of cilium genes in the EPC-fate specific genes. (D) Top Go terms of biological processes enriched in 869 non-ciliary EPC-fate specific genes. (E) Expression profiles of hydrocephalus genes and EPC-fate regulators in the bifurcating trajectory. (F) Quantification results for Fig 4G. Three independent experiments were performed. Error bars represent SD. Asterisks indicate P-values from Student’s t-tests, *P < 0.05; **P < 0.01; ***P < 0.001; ns, not significant. The data underlying this figure can be found at S1 Data, specifically in the sheet labeled ‘S4 Fig’. (PDF) [file pbio.3003318.s004.pdf]

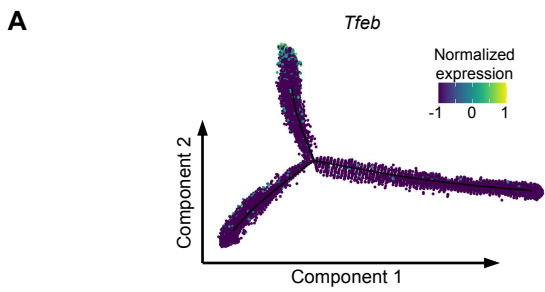

**B**

| Celltype   | Expression |
|------------|------------|
| Glioblast  | 0.04       |
| EPC        | 0.27       |
| Neuroblast | 0.01       |
| Neuron     | 0.00       |

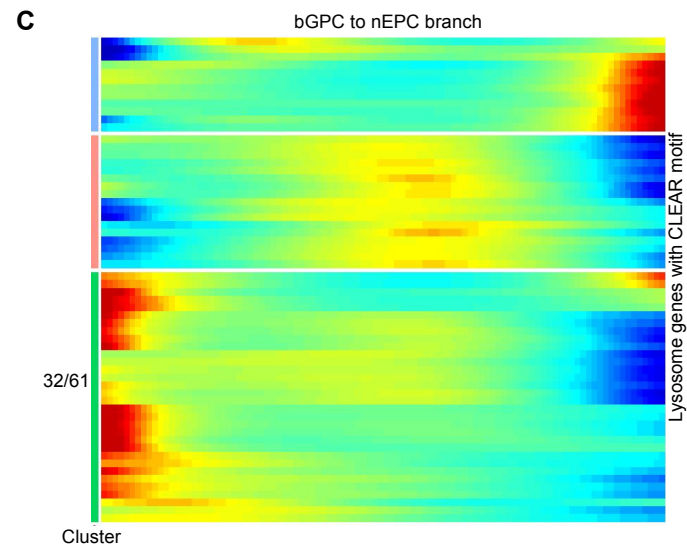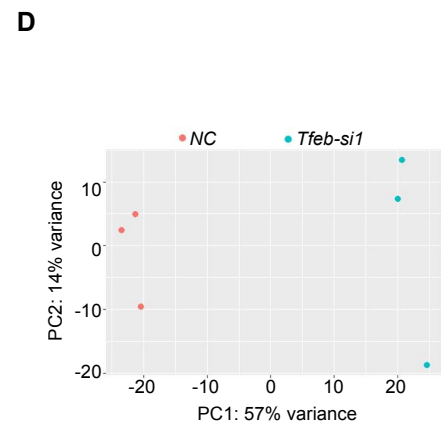

**Supplementary Figure 6**

Supplement: S6 Fig — (A) Expression profile of Tfeb along the bifurcating trajectory. (B) Expression values of Tfeb in the indicated cell types of the developing mouse brain dataset. (C) Heatmap shows the dynamic expression of lysosome genes with CLEAR motif along the nEPC branch. (D) Principal component analysis of three replicates of non-targeting control and Tfeb-si1 samples from the bulk RNA-seq experiments. (PDF) [file pbio.3003318.s006.pdf]

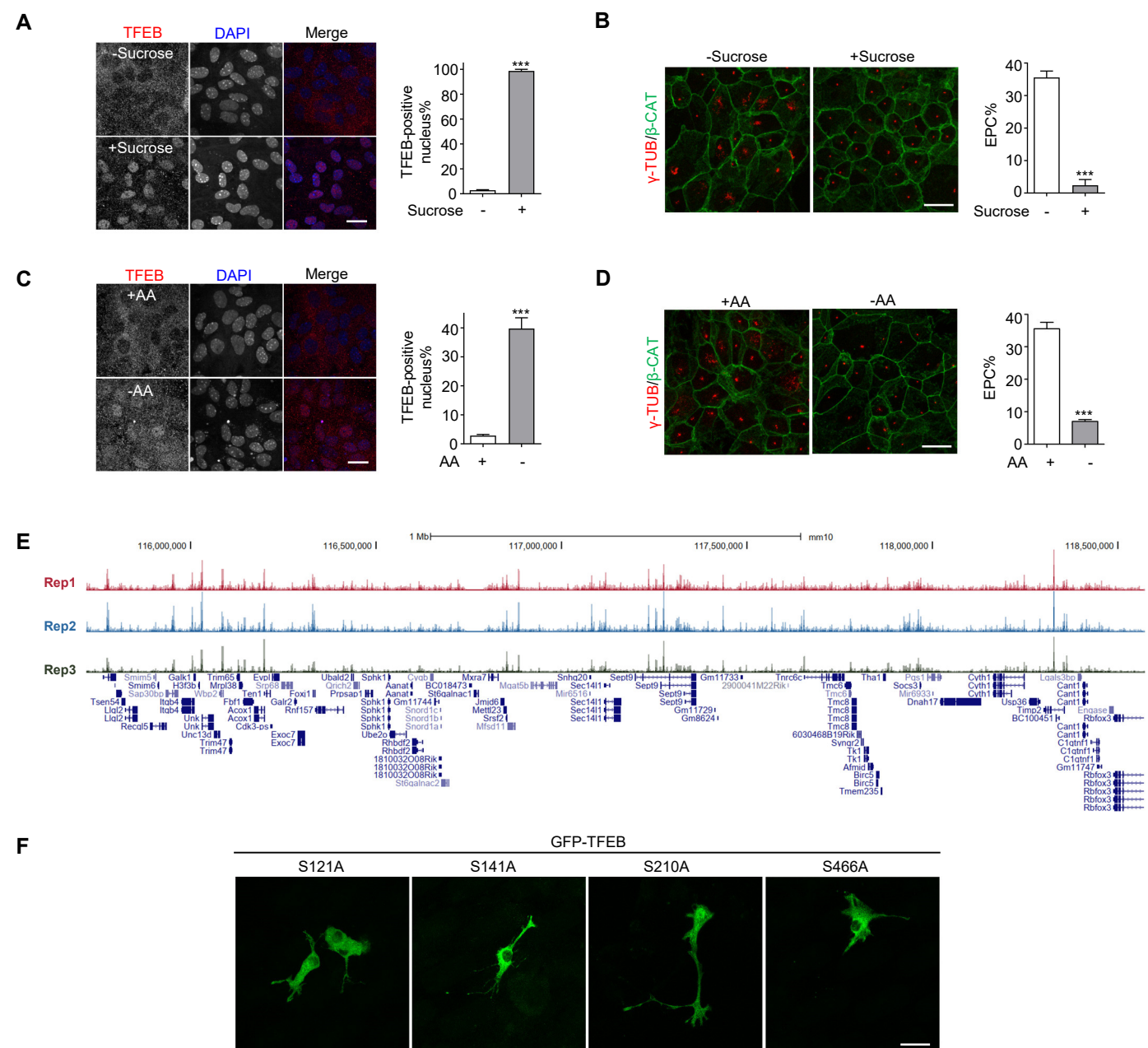

### Supplementary Figure 8

Supplement: S8 Fig — (A) TFEB translocated from cytosol to nucleus after 100 mM sucrose treatment. At least 151 cells were quantified in each experiment and condition. (B) A dramatic reduction in the EPC percentage after 100 mM sucrose treatment. At least 233 cells were scored in each experiment and condition. (C) Amino acid starvation induced translocation of TFEB from the cytosol to the nucleus. At least 151 cells were quantified in each experiment and condition. (D) EPC-lineage differentiation was drastically suppressed after amino acid starvation. At least 233 cells were scored in each experiment and condition. All of the quantification results above were from three independent experiments. Error bars represent the SD. Asterisks indicate P-values from Student’s t-tests, ***P < 0.001. The scale bars above are 20 μm. (E) UCSC genome browser track showing the good quality of the TFEB ChIP-seq data. (F) Among the four reported consecutive active mutants of TFEB, only TFEB-S210A showed predominant nuclear localization when transfected into GPCs. The scale bar is 25 μm. The data underlying this figure can be found at S1 Data, specifically in the sheet labeled ‘S8 Fig’. (PDF) [file pbio.3003318.s008.pdf]

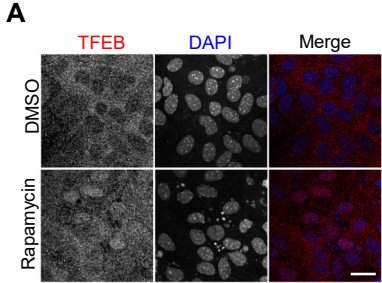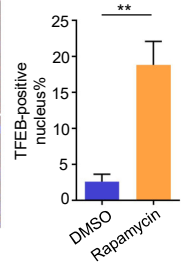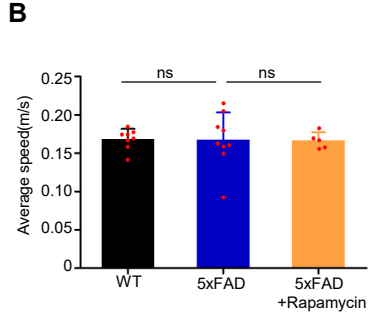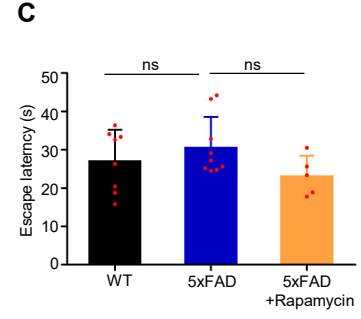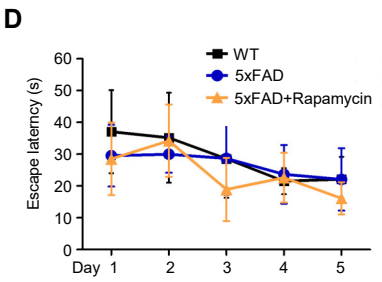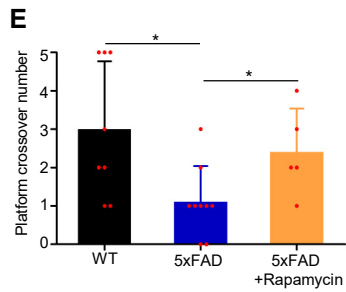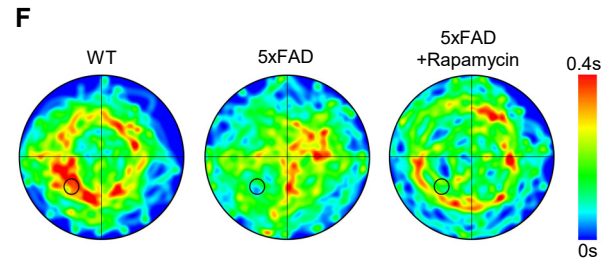

**Supplementary Figure 9**

Supplement: S9 Fig — (A) Immunofluorescence analyses showing the translocation of TFEB from the cytosol to the nucleus upon 5 μM Rapamycin treatment. The quantification results were from three independent experiments. At least 288 cells were scored in each experiment and condition. The scale bar is 20 μm. (B) Quantification of moving speed of 3-month-old mice in each group during visible platform trail. (C) Quantification of escape latencies during visible platform trail. (D) Quantification of escape latencies during 5-day hidden platform trail. (E) Quantification of platform crossover number during probe trial. (F) Average movement heatmap of mice in each group during probe trial. The circle indicates the platform position. 5–9 mice were used for each group. Error bars represent SD. Asterisks indicate P-values from Student’s t-tests, *P < 0.05; **P < 0.01; ns, not significant. The data underlying this figure can be found at S1 Data, specifically in the sheet labeled ‘S9 Fig’. (PDF) [file pbio.3003318.s009.pdf]
