# Supplementary material for: Lineage trajectories and fate determinants of postnatal neural stem cells and ependymal cells in the developing ventricular zone
Source: PLoS Biol. 2025 Jul 30;23(7):e3003318. doi: 10.1371/journal.pbio.3003318 (PMC12327645; doi:10.1371/journal.pbio.3003318)
Supplement: S3 Fig — (A) Top 10 GO terms of biological processes from genes differentially expressed in the two clusters along the nEPC branch. (B) Heatmap showing the dynamic expression of multicilia-specific genes along the nEPC branch. (C) The four multicilia-specific genes absent in our data were also not detectable in the adult VZ scRNA-seq and ependymal cilia proteomic data. (PDF) [file pbio.3003318.s003.pdf]

A

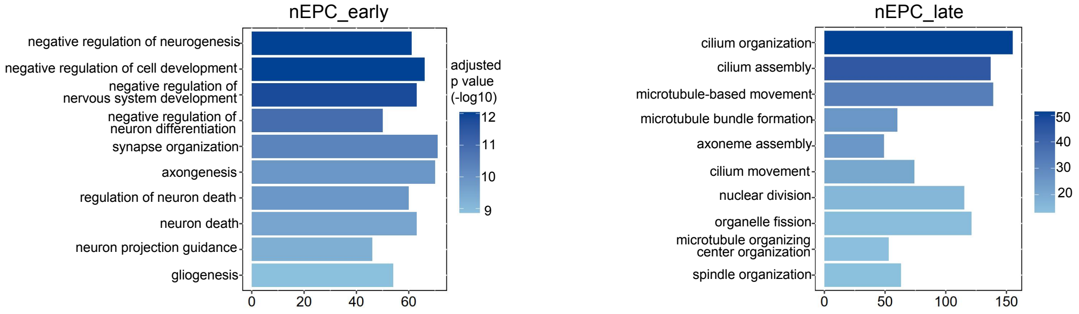

B

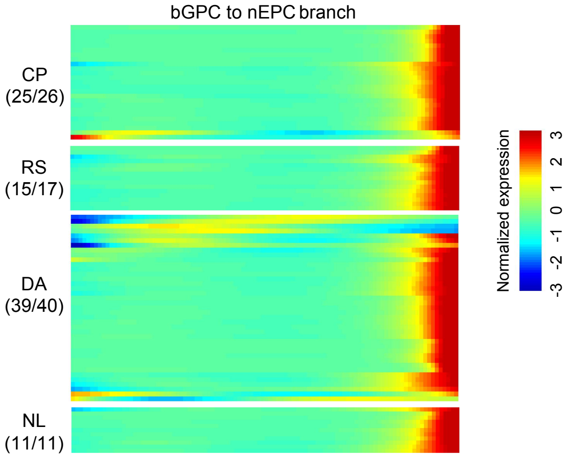

C

Gene	Structure	Adult VZ scRNA-seq	Ependymal cilia proteomics
**Atp2b3**	Central pair	Absent	Absent
**Calm4**	Radial spoke	Absent	Absent
**Rsph6a**	Radial spoke	Absent	Absent
**Cetn1**	Dynein arm	Absent	Absent

Supplementary Figure 3
