# Supplementary material for: Lineage trajectories and fate determinants of postnatal neural stem cells and ependymal cells in the developing ventricular zone
Source: PLoS Biol. 2025 Jul 30;23(7):e3003318. doi: 10.1371/journal.pbio.3003318 (PMC12327645; doi:10.1371/journal.pbio.3003318)
Supplement: S5 Fig — (A and C) Expression profiles of Npas1 (A) and Foxa2 (C) along the bifurcating trajectory. (B and D) Expression values of Npas1 (B) and Foxa2 (D) in the indicated cell types of the developing mouse brain dataset. (E) Immunoblotting showed efficient depletion of Foxj1 in EPCs by RNAi. siRNA transfection was performed at SS d − 1 and SS d + 2 then cells were harvested at SS d + 5. The quantification results were from three independent experiments. Error bars represent SD. Asterisks indicate P-values determined by Student’s t-tests between NC and siRNA-treated groups, ***P < 0.001. The data underlying this figure can be found at S1 Data, specifically in the sheet labeled ‘S5 Fig’. (PDF) [file pbio.3003318.s005.pdf]

A

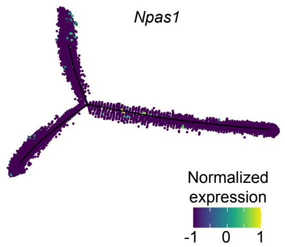

B

Celltype	Expression
Glioblast	0.02
EPC	0.19
Neuroblast	0.00
Neuron	0.02

C

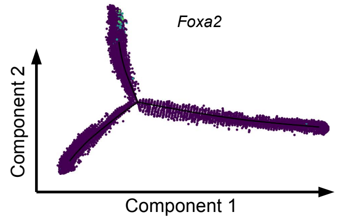

D

Celltype	Expression
Glioblast	0.01
EPC	0.20
Neuroblast	0.02
Neuron	0.02

E

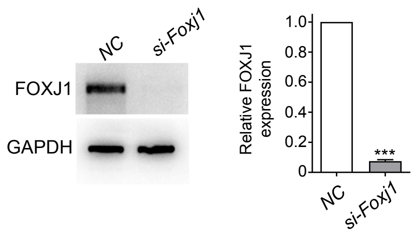

Supplementary Figure 5
