# Supplementary material for: Lineage trajectories and fate determinants of postnatal neural stem cells and ependymal cells in the developing ventricular zone
Source: PLoS Biol. 2025 Jul 30;23(7):e3003318. doi: 10.1371/journal.pbio.3003318 (PMC12327645; doi:10.1371/journal.pbio.3003318)
Supplement: S1 Table — (DOCX) [file pbio.3003318.s010.docx]

**S1 Table. List of antibodies used for immunoblotting (IB), immunofluorescence (IF) or immunoprecipitation (IP)**

| **Antigen** | **Species** | **Supplier** | **Catalog no.** | **IB** | **IF** | **IP** |
| --- | --- | --- | --- | --- | --- | --- |
| CD133-PE | Rat | eBioscience | 12-1331-82 |  | 1:100 |  |
| ARL13B | rabbit | Proteintech Group | 17711-1-AP | 1:2000 | 1:1000 |  |
| Ki67 | mouse | BD | 550609 |  | 1:50 |  |
| acetylated-tubulin | mouse | Santa Cruz | sc-23950 | 1:2000 | 1:400 |  |
| ASCL1 | rabbit | Abcam | ab211327 |  | 1:100 |  |
| TUBB3 | rabbit | Sigma-Aldrich | T2200 |  | 1:200 |  |
| PDGFRA | mouse | Santa Cruz | sc-398206 |  | 1:100 |  |
| NKX2-2 | rabbit | Abcam | ab191077 |  | 1:100 |  |
| OLIG2 | rabbit | Proteintech Group | 13999-1-AP | 1:2000 | 1:400 |  |
| DCX | mouse | Santa Cruz | sc-271390 |  | 1:100 |  |
| FOXJ1 | mouse | Santa Cruz | sc-53139 |  | 1::100 |  |
| FOXJ1 | mouse | Thermo | 14-9965 | 1:500 |  |  |
| LHX2 | rabbit | Abcam | ab184337 | 1:2000 |  |  |
| GAPDH | mouse | ABclonal | AC002 | 1:5000 |  |  |
| γ-tubulin | rabbit | Sigma-Aldrich | T5192 | 1:5000 | 1:1000 |  |
| β-catenin | mouse | BD | 610153 |  | 1:500 |  |
| TFEB | rabbit | Bethyl Laboratories | A303-673A | 1:2500 |  | 1:250 |
| TFEB | rabbit | Proteintech Group | 13372-1-AP |  | 1:200 |  |
| LAMP1 | rabbit | Proteintech Group | 21997-1-AP | 1:1000 |  |  |
| LAMP2 | mouse | Proteintech Group | 66301-1-Ig | 1:1000 |  |  |
| FLAG | mouse | Sigma-Aldrich | F1804 | 1:5000 |  | 1:500 |
| GFP | rabbit | Proteintech Group | 50430-2-AP | 1:2000 |  |  |
| GFP | chicken | Abcam | ab13970 |  | 1:2000 |  |
| anti-Mouse IgG (H+L)-HRP | goat | Thermo | G-21040 | 1:5000 |  |  |
| anti-Rabbit IgG (H+L)-HRP | goat | Thermo | G-21234 | 1:5000 |  |  |
| anti-Rabbit IgG (H+L)-Alexa Fluor 488 | donkey | Jackson ImmunoResearch | 715-545-150 |  | 1:1000 |  |
| anti-Rabbit IgG (H+L)-Cy3 | donkey | Jackson ImmunoResearch | 711-165-152 |  | 1:1000 |  |
| anti-Chicken IgY (H+L)- Alexa Fluor 488 | goat | Thermo | A11039 |  | 1:1000 |  |
| anti-Rabbit IgG (H+L)- Alexa Fluor 647 | donkey | Jackson ImmunoResearch | 711-605-152 |  | 1:1000 |  |
