# Supplementary material for: Lineage trajectories and fate determinants of postnatal neural stem cells and ependymal cells in the developing ventricular zone
Source: PLoS Biol. 2025 Jul 30;23(7):e3003318. doi: 10.1371/journal.pbio.3003318 (PMC12327645; doi:10.1371/journal.pbio.3003318)
Supplement: S2 Table — (DOCX) [file pbio.3003318.s011.docx]

**S2 Table. List of siRNA sequences**

| Name | Sequence (5’ to 3’) |
| --- | --- |
| *NC* | UUCUCCGAACGUGUCACGUtt |
| *Mcidas-si1* | GCGUCUCCUAGUGGUGAUUtt |
| *Mcidas-si2* | GGUGGUUACAAAUUCCGCUtt |
| *Lhx2-si1* | GGAUGGCAGCAUCUACUGCtt |
| *Lhx2-si2* | GUAGACAAGCAAUGGCACAtt |
| *si-Npas1* | GGUCCUUCUUUGUCCGCAUtt |
| *si-Foxj1* | CUCACAUGGAGAUGUGGAUCUUA |
| *si-Foxa2* | CGCGCUCGGGACCCCAAGAtt |
| *Tfeb-si1* | GCGAGAGCUAACAGAUGCUtt |
| *Tfeb-si2* | GGAUCAAGGAGCUGGGAAUtt |
| *si-Gmnc* | CCAUUGAUGACACUCUACCAGCUAA |
| *Gmnn-si1* | AGCCUUUGAUCUUAUAAGUtt |
| *Gmnn-si2* | UCGAGAGGCUGAGUAAUGAtt |
