# Supplementary material for: Lineage trajectories and fate determinants of postnatal neural stem cells and ependymal cells in the developing ventricular zone
Source: PLoS Biol. 2025 Jul 30;23(7):e3003318. doi: 10.1371/journal.pbio.3003318 (PMC12327645; doi:10.1371/journal.pbio.3003318)
Supplement: S3 Table — (DOCX) [file pbio.3003318.s012.docx]

**S3 Table. List of plate-based scRNA-seq primer sequences**

| Name | Sequence (5’ to 3’) |
| --- | --- |
| Barcoded RT primer | CTACACGACGCTCTTCCGATCT [8-bp well barcode] NNNN  NNNNNNTTTTTTTTTTTTTTTTTTTTTTTTTVN |
| Template Switching Oligo | AAGCAGTGGTATCAACGCAGAGTACATrGrGrG |
| cDNA PCR Forward Primer | CTACACGACGCTCTTCCGATCT |
| cDNA PCR Reverse Primer | AAGCAGTGGTATCAACGCAGAG |
| Library PCR Universal Primer | AATGATACGGCGACCACCGAGATCTACACTCTTTCCCTACACGACGCTC |
| Library PCR Index Primer | CAAGCAGAAGACGGCATACGAGAT [8-bp plate barcode] GTCTCGTGGGCTCGG |
