# Supplementary material for: Lineage trajectories and fate determinants of postnatal neural stem cells and ependymal cells in the developing ventricular zone
Source: PLoS Biol. 2025 Jul 30;23(7):e3003318. doi: 10.1371/journal.pbio.3003318 (PMC12327645; doi:10.1371/journal.pbio.3003318)
Supplement: S4 Table — (DOCX) [file pbio.3003318.s013.docx]

**S4 Table. List of qPCR primer sequences**

| Name | Sequence (5’ to 3’) |
| --- | --- |
| Mcidas-F | AACCGAAGCGTCTCCTAGTG |
| Mcidas-R | GGTCATCCATTGCATCTCTG |
| Npas1-F | TCTTCGACTACATCCATCCT |
| Npas1-R | TCACATGAATGACCTTGTACC |
| Foxa2-F | CCCTACGCCAACATGAACTCG |
| Foxa2-R | GTTCTGCCGGTAGAAAGGGA |
| Gapdh-F | GACAACTTTGGCATTGTGGA |
| Gapdh-R | ATACTTGGCAGGTTTCTCCAG |
| Gmnc-F | GAAATAAACAGCTCCAGGATACTC |
| Gmnc-R | CTGTGTTCTTTGGGTTTCCTC |
| Gmnn-F | AGCCTTCTGCAGATGGATCTC |
| Gmnn-R | CTCAGCTACTTCTGCCAAGTC |
| Tfeb-F | CAACAGTCCCAGCATCAGAA |
| Tfeb-R | GGCGCATAATGTTGTCAATG |
