# Supplementary material for: Lineage trajectories and fate determinants of postnatal neural stem cells and ependymal cells in the developing ventricular zone
Source: PLoS Biol. 2025 Jul 30;23(7):e3003318. doi: 10.1371/journal.pbio.3003318 (PMC12327645; doi:10.1371/journal.pbio.3003318)
Supplement: S1 Raw Images — (PDF) [file pbio.3003318.s015.pdf]

Raw images of immunoblots in Figure 4G

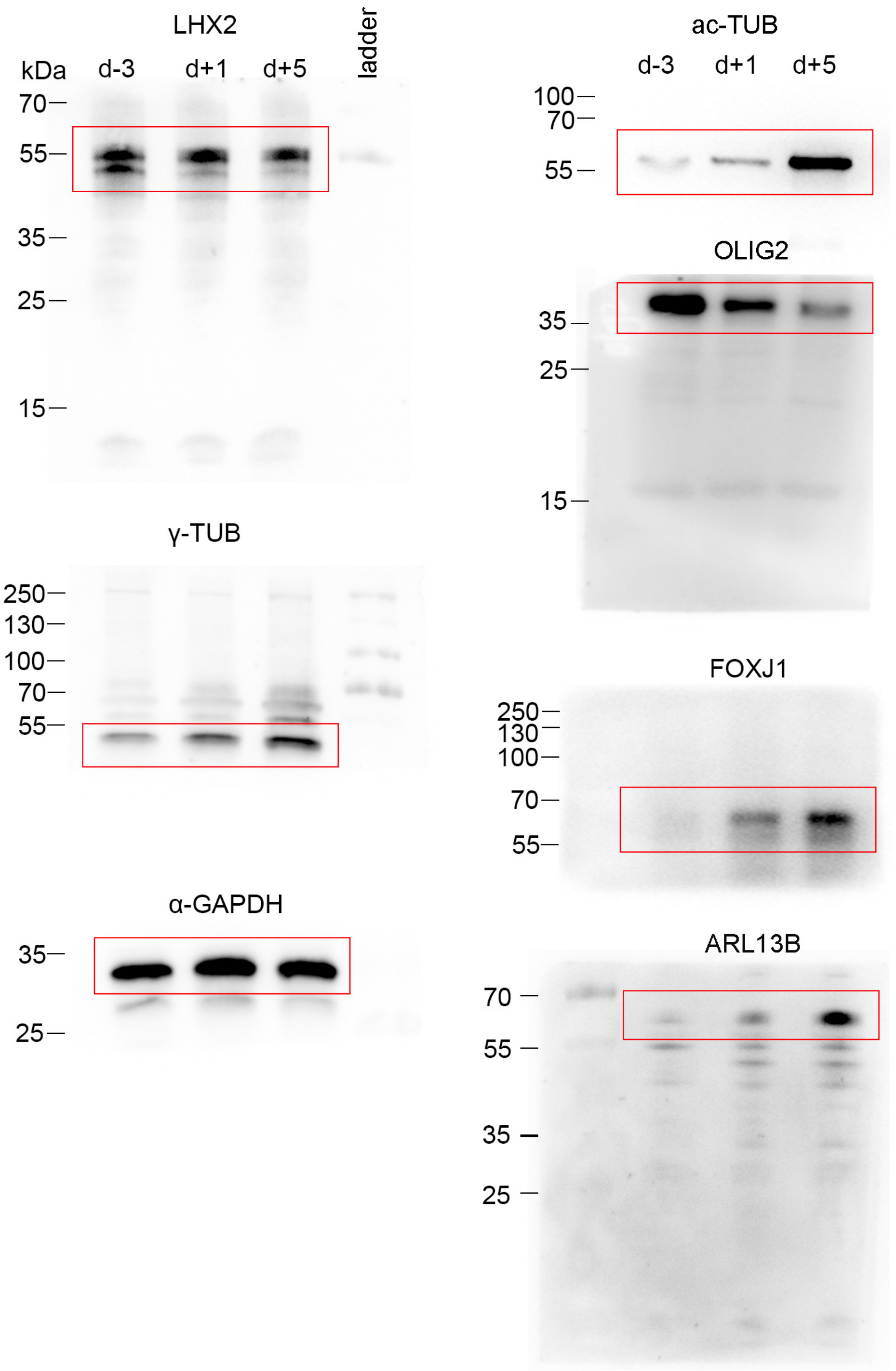

Immunoblots were revealed using the Clarity Western ECL Substrate kit. Images were taken using Tanon 5200 Chemiluminescent Imaging System. Cropped images shown in Figure 4G are indicated by red rectangles.

Raw images of immunoblots in Figure 5C

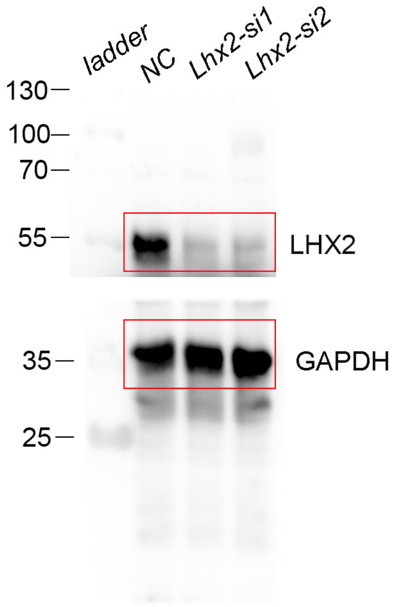

Immunoblots were revealed using the Clarity Western ECL Substrate kit. Images were taken using Tanon 5200 Chemiluminescent Imaging System. Cropped images shown in Figure 5C are indicated by red rectangles.

Raw images of immunoblots in Figure 6B and C

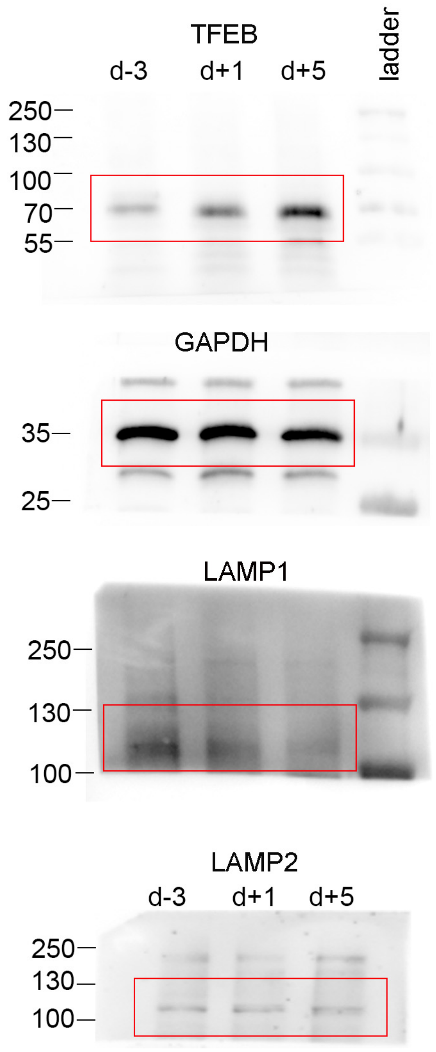

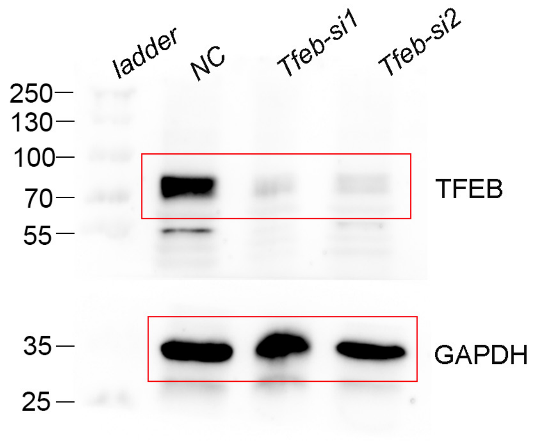

Immunoblots were revealed using the Clarity Western ECL Substrate kit. Images were taken using Tanon 5200 Chemiluminescent Imaging System. Cropped images shown in Figure 6B and C are indicated by red rectangles.

Raw images of immunoblots in Figure 7G

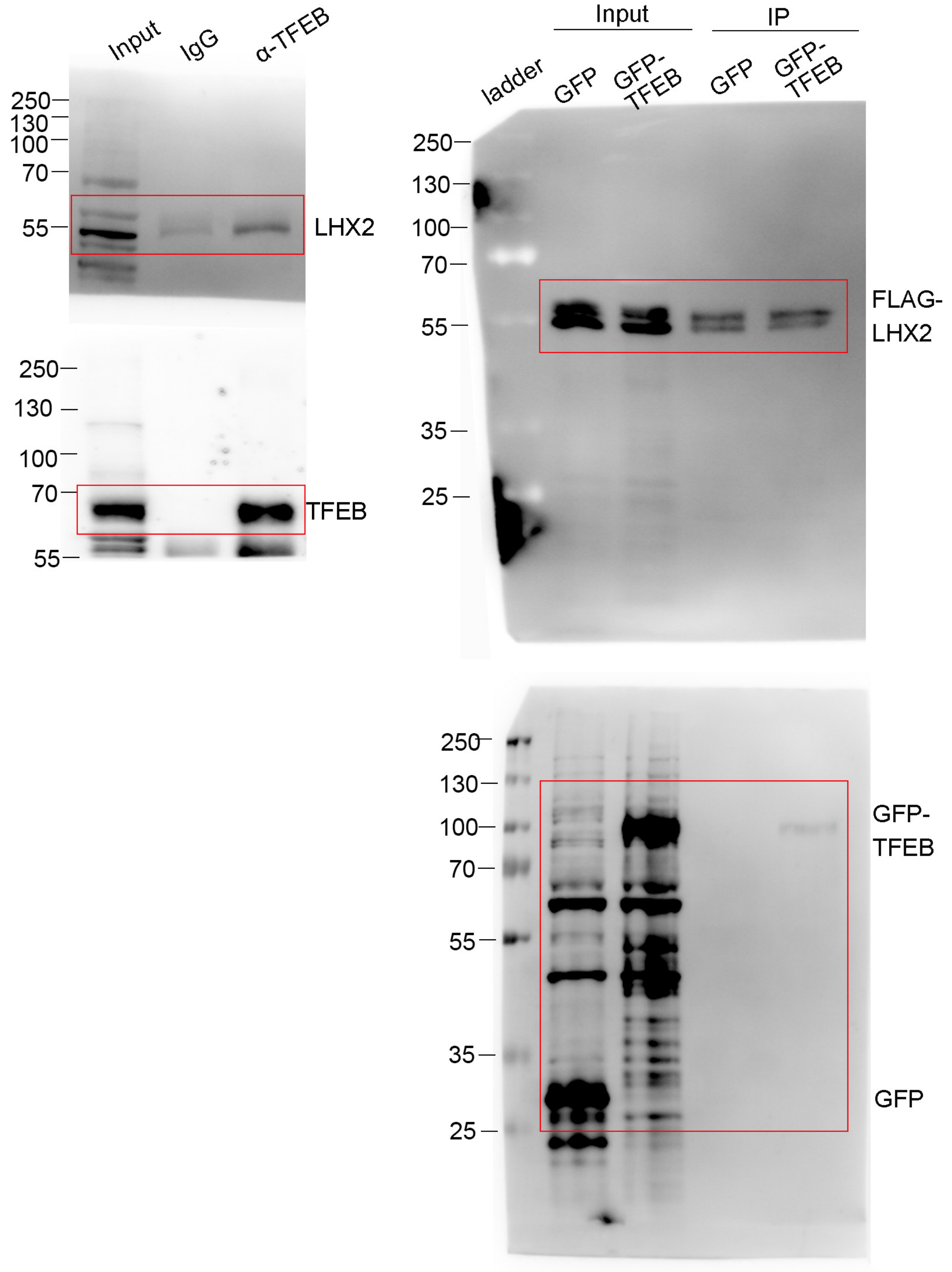

Immunoblots were revealed using the Clarity Western ECL Substrate kit. Images were taken using Tanon 5200 Chemiluminescent Imaging System. Cropped images shown in Figure 7G are indicated by red rectangles.

Raw images of immunoblots in Supplementary Figure 5E

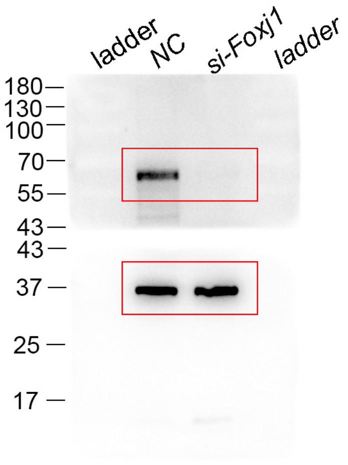

Immunoblots were revealed using the Clarity Western ECL Substrate kit. Images were taken using Tanon 5200 Chemiluminescent Imaging System. Cropped images shown in Supplementary Figure 5E are indicated by red rectangles.

Raw images of immunoblots in Supplementary Figure 7E

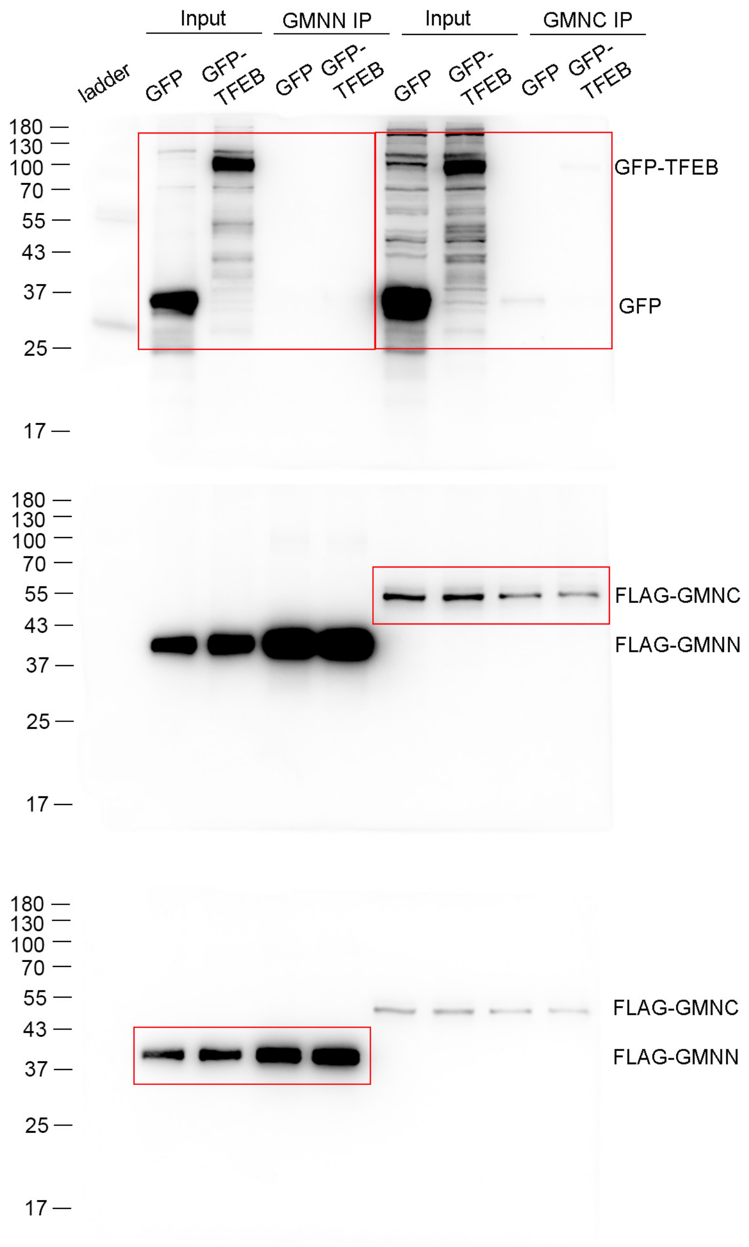

Immunoblots were revealed using the Clarity Western ECL Substrate kit. Images were taken using Tanon 5200 Chemiluminescent Imaging System. Cropped images shown in Suppementary Figure 7E are indicated by red rectangles.
