# Supplementary material for: Lineage trajectories and fate determinants of postnatal neural stem cells and ependymal cells in the developing ventricular zone
Source: PLoS Biol. 2025 Jul 30;23(7):e3003318. doi: 10.1371/journal.pbio.3003318 (PMC12327645; doi:10.1371/journal.pbio.3003318)
Supplement: Attachment — Submitted filename: Response to Reviewers_250509.pdf [file pbio.3003318.s019.pdf]

Response to reviewers' comments

We thank both reviewers for their comments and constructive feedback, which has offered the opportunities for us to make improvements on the manuscript. We provide below a point-by-point response to address the concerns raised by the reviewers.

Black – reviewers' comments
Blue – our responses to reviewers' comments
Red – changes made to the original manuscript

Reviewer #1:
The paper has interesting findings summarised below:
- There are three distinct cellular states in the developing ventricular zone forming a continuous bifurcating trajectory (bGPCs, nNSCs-NBs, n-EPCs).
- A novel intermediate state of cells expressing markers of oligodendrocytes and neuroblasts was detected.
- Transcriptional factors essential for ependymal cell lineage differentiation were identified.
- TFEB was pointed out as a master regulator, tuning neural stem cell and ependymal cell bifurcation, in cooperation with LHX2.

1. The authors unravel parts of the molecular mechanism underlying cell fate decisions of progenitor cells towards neural stem or ependymal cell fates. As the common origin of NSCs and ECs have been previously described and the involvement of Geminin family members in this decision it would be required the authors to show which is the cooperation of TFEB with the pathway regulated by Geminin and GemC1.

We thank the reviewer for the comments. To investigate the relationship between TFEB and Geminin family members, we performed *Gmnc* (GemC1) knockdown, *Gmnn* (Geminin) knockdown and co-immunoprecipitation analysis. In brief, we found that TFEB undergoes indirect modulation by GemC1 in the process of EPC specification. The related results were presented in Supplementary Figure 7A-E shown below:

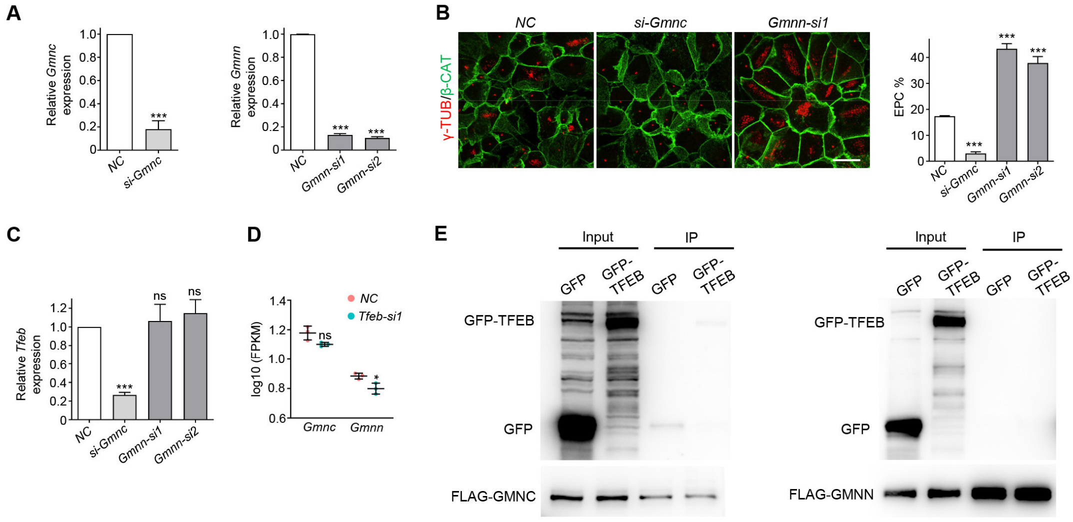

Supplementary Figure 7

And the following text describing the results was included in the Results section (page 19-20, line 404-419):

Previous studies have demonstrated that the antagonistic Geminin family members *Gmnn* and *Gmnc* modulate RGC fate decision. To investigate the interplay between TFEB and the Geminin members, siRNA-mediated knockdown of *Gmnc* or *Gmnn* was performed in the *in vitro* culture systems (**Supplementary Figure 7A**). In accordance with their known roles, *Gmnc* deficiency blocked EPC-lineage commitment, whereas *Gmnn* depletion increased the EPC population (**Supplementary Figure 7B**). QPCR analysis showed that *Gmnc* ablation caused a 73.5% reduction in *Tfeb* expression, while the loss of *Gmnn* exerted no significant effect (**Supplementary Figure 7C**). This finding is consistent with a recent published dataset displaying that GMNC overexpression induced upregulated expression of *Tfeb*. On the other hand, the loss of TFEB did not impact *Gmnc* expression and slightly reduced *Gmnn* expression by 17.8% (**Supplementary Figure 7D**). Moreover, co-immunoprecipitation assays revealed the absence of direct physical interactions between TFEB and either Geminin protein (**Supplementary Figure 7E**). These results suggest that TFEB undergoes indirect modulation by GMNC during multiciliogenesis.

2. Introduction details of the already known molecular pathway regulating cell fate decisions of ependymal cells should be added as there are used later in the text.

We thank the reviewer for the suggestion. We agree adding current knowledge on molecular pathway regulating cell fate decisions of ependymal cells will make the flow easier to read, and they have been added to the Introduction section as suggested (page 4, line 68-74), which is also shown below:

Motile ciliogenesis during EPC differentiation is a multi-step process orchestrated by a tightly regulated transcriptional program. Upstream of the ciliogenesis pathway, GMNC and MCIDAS serve as primary regulators governing centriole amplification, a process essential for the assembly of multiple cilia. Downstream transcription factors (TFs) FOXJ1 and RFX family members function as direct regulators of core ciliogenic genes. MYB and TRP73 have also been identified as critical participants in EPC differentiation process.

3. CD133 is also expressed in neural stem cells and in mature ependymal cells constituting a problem in previous studies when trying to discriminate neural stem cells and ependymal cells. How authors have verified that CD133 is specifically expressed in radial cells at that time point you're performing the experiments and you are not including populations of neural stem or ependymal cells in your analysis?

We thank the reviewer for raising this point. Radial glial cells (RGCs) are named after their uniform radial morphology, harbouring significant molecular and functional heterogeneity[1]. Prior studies established that RGCs occupied VZ at the timepoints we selected (P0 and P5)[2] and RGCs fulfilled differentiation into EPCs around P15[3]. To validate this timeline, we performed immunofluorescence staining to examine the CD133 expression pattern and cellular heterogeneity of RGCs at P0, P5 and P15. Consistent with previous findings, CD133 localized specifically to the apical surface of RGCs at all examined timepoints. These

results have been added as the new Figure 1A and B in the current version of the manuscript shown below:

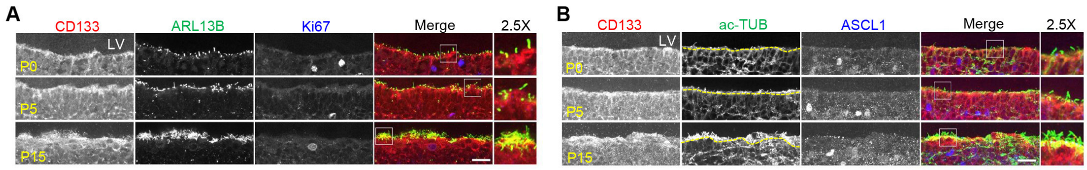

Labeling of the ciliary membrane marker (ARL13B) and the ciliary microtubule marker (ac-TUB) revealed progressive ciliogenesis in CD133-positive RGCs: cells extended singular short primary cilia at P0, formed both primary cilium and multicilia by P5, and manifested dense multicilia by P15. The dynamic ciliogenesis pattern excluded mature EPCs from our P0 and P5 analysis. In agreement, cells expressing PEDF1, a trophic factor secreted by mature EPCs[4], were nearly absent in our scRNA-seq data (Reviewer Figure 1). For NSC lineage analysis, immunostaining of cell proliferating marker (Ki67) and NSC marker (ASCL1) implied that some CD133-positive RGCs have acquired transcriptional characteristic of NSCs by P0. Consistently, some cells identified in our scRNA-seq data were found to express NSC markers including *Ascl1*, *Egfr* and *Sox11* (Figure 1F). Thus, the RGCs we sorted by CD133 labeling were constituted of three terminal cell types, which were bipotent glial progenitor cells (bGPCs), immature EPCs with sparse mulitcilia (nEPCs) and nNSC-neuroblasts, together with cells in transitional states along the differentiation continuum. Despite harbouring high heterogeneity, all of these CD133-positive cells were classified as RGCs at this stage due to their defining uniform radial morphology.

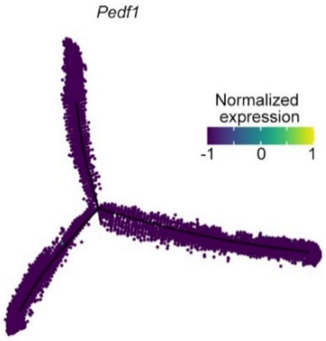

Reviewer Figure 1

4. Percentages from quantifications are not provided in all graphs and should be added.

We thank the reviewer for the suggestion. We have added the quantification results to Figures 2F, 4G (presented in Supplementary Figure 4F), 5C, 6B-C, 7A, 7C, 7D and Supplementary Figures 5E, 8A, 8C in the revised manuscript.

5. Discussion needs to be more general and not so focused on the figures that you have analysed before in the results section.

This is a good suggestion. We have modified the Discussion section accordingly as requested.

6. Please check that you refer to the right figures when trying to support your statements.

We thank the reviewer for the careful reading, and we have now proofread the manuscript to make sure we refer to the right figures.

7. In the section describing immunofluorescent staining of cultured cells/ tissue sectioning and immuno-staining/ immunoblotting / immunoprecipitation, antibodies/antibody-bound beads calalogue numbers and used dilutions are not mentioned and should be included. Issue the way that qualifications were done should be explained (How many fields and cells were counted and how statistics were performed).

We thank the reviewer for raising these problems and apologise for the omission of the information. Calalogue numbers and dilutions of antibodies used for immuno-staining/ immunoblotting/immunoprecipitation can now be found in Supplementary Table 1. Information of beads are provided in the Methods section (page 43, line 918-919). The ways of quantifications have been summarized in the Methods section (page 39, line 822-826 and page 40, line 844-845), which is also shown below:

For Figures 5-7 and Supplementary Figures 7-9, at least 151 cells from 6 images were quantified in each experiment and condition. For Figure 8, at least 64 GFP-positive cells from 43 images were quantified in each experiment and condition. Two-sided Student's t test was used to calculate P-values between unpaired samples.

For Figure 2F, at least 164 cells from 6 images of P0 brain sections were quantified in each experiment.

8. The immunofluorescence figures are of poor quality the positive cells cannot be discriminated from the background.

Thanks for pointing out the problem. We have replaced the old immunofluorescence images of poor quality in Figures 2F, 7C-D and Supplementary Figures 2B, 7A, 7C (Supplementary Figures 2C, 8A, 8C in the revised manuscript). And now the positive cells can be easily discriminated from the background. The figures were also shown below:

Figure 2F

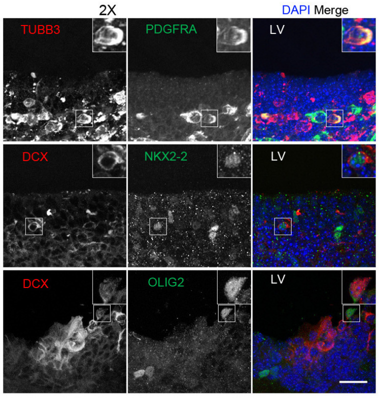

Supplementary Figure 2B

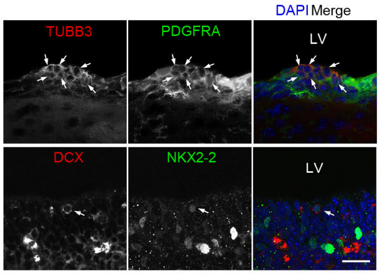

Figure 7C

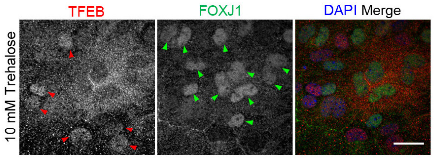

Figure 7D

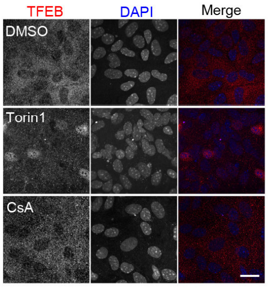

Supplementary Figure 8A

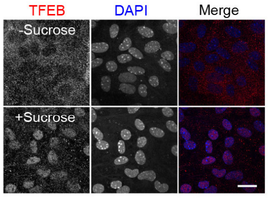

Supplementary Figure 8C

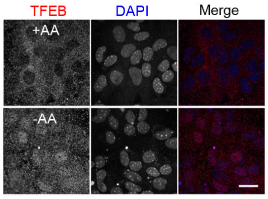

9. The ependymal culture protocol in which you are referring never obtains neuronal cells can be specify whether you have made any alterations to the protocol.

We thank the reviewer for the comment and sorry about the confusion. The experimental scheme of EPC culture is shown in Figure 4F in the revised manuscript. We denoted the day when serum starvation was applied as day 0. Steps that happened during the days before the serum starvation were denoted with negative numbers, and those after the serum starvation positive numbers. RGCs were isolated from neonatal VZs and plated in 25cm^2 flasks at day -5. After culturing for 2 days (on day -3), neurons were shaken off and removed. The remaining RGC-enriched cells were transferred into glass-bottom dishes at a density of 5x10^5 cells/cm^2 (approximately 90% confluency) on day -2. RGCs further proliferate and attain 100% confluency on day 0. Then cells were subject to serum starvation to induce EPC-lineage differentiation. The procedures of vigorous shaking on day -3 and serum starvation starting from day 0 till the end can eliminate majority of neurons and neuroblasts.

Minor comments

line 69: instead of rhythmic beating use the term coordinated

We have made the change accordingly in the revised manuscript (page 4, line 76).

line 73: disruption of the circulation of CSF is one of the causes that can possibly lead to hydrocephalus development- there are also other reasons that could results to hydrocephalus

We have modified the sentence to 'Disruption of the circulation of CSF can possibly lead to hydrocephalus development' (page 4, line 79-80).

line 233-236: it is proposed that ciliary genes may be implicated in bGPC-nNSC differentiation. Do all bGPCs express ciliary markers and later on there is mechanism that regulates their cell fate towards NSCs or ECs? Or this is a distinct subpopulation of bGPCs from the one you mentioned before expressing qNSC markers?

Our analysis revealed that ciliary genes were only expressed in some cells of bGPC cluster (Reviewer Figure 2A), displaying significantly lower cellular prevalence but comparable expression levels relative to nEPC_late cluster (Reviewer Figure 2B). These ciliary gene positive cells were embedded within the bGPC cluster and didn't form a transcriptionally distinct subpopulation (Reviewer Figure 2A). Given EPCs greatly outnumber NSCs in the adult VZ[5], we proposed that these bGPCs maintain ciliary gene expression as a lineage-priming mechanism to bias differentiation towards EPC fates while actively suppressing NSC overproduction.

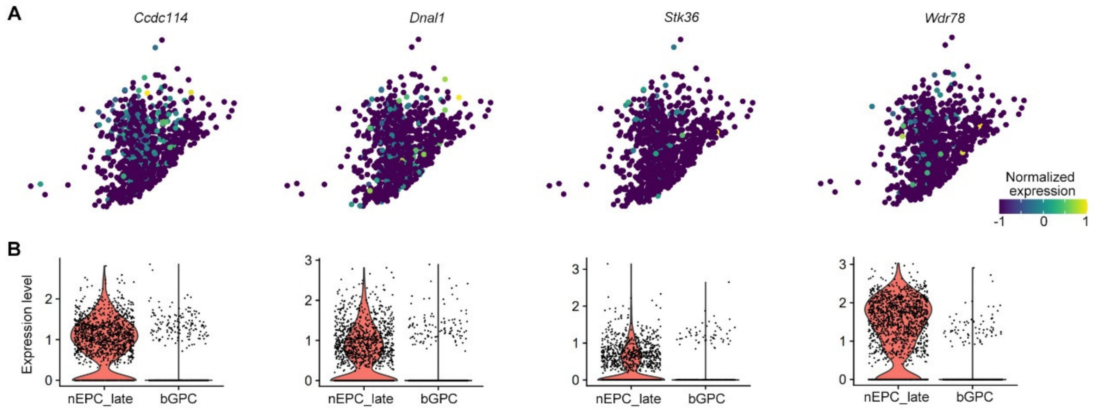

Reviewer Figure 2

line 251: use hydrocephalus development than formation

We have made the change as suggested (page 14, line 283).

line 256: beating of cilia is the main propelling force for CSF circulation in mice. So it should be clarified because this does not apply for humans

Thanks for pointing this out. We have added 'in mice' to the sentence to improve the clarity (page 14, line 288).

line 271: the word Six- I guess you mean Six3

Sorry for the mistake. We have corrected it in the revised manuscript (page 15, line 302).

line 299: You refer to figure 4F but this is not the right figure

Sorry for the mistake. The experimental scheme of EPC culture has been included to Figure 4F in the revised manuscript.

line 313-314: It is mentioned that "loss of LhX2 promoted EPC-lineage specification (fig 5A, B) which agreed with the in vivo results reported later".
In which in vivo experiments do you refer to?

The *in vivo* experiments we refer to were performed in the cited paper[6] (ref 56 in the current version of the manuscript). Utilizing of *in situ* hybridization assay combined with ultrastructural analysis, Salvatierra and colleagues revealed that *Lhx2*-deficient tanycytes lost expression of tanycyte-specific genes and became multiciliated ependymal cells *in vivo* (see Figure 3 and Figure 5 in the reference [6]).

In fig.4E is mentioned that reduction of LhX2 is related to NSC formation, - we would expect ependymal cells to be reduced and have an increase in neural stem cells in fig 5 as well.

Sorry for the confusion. We didn't mention that reduction of Lhx2 is related to NSC formation. The even expression in the nEPC branch and down-regulation in the nNSC-NB branch of *Lhx2* resulted in its relatively increased expression along nEPC branch compared to nNSC-NB branch (Figure 4E). The expression kinetics of *Lhx2* was supposed to regulate EPC production, and we termed the TFs presented in Figure 4D and 4E 'EPC-fate regulators' (page 15, line 304). Consistent with prior studies demonstrating the inhibitory role in EPC specification[6], our ChIP-Seq and immunoprecipitation analyses revealed a mechanistic pathway whereby LHX2 suppresses ciliogenic gene expression through interaction with TFEB (Figure 7F-I). In Figure 5C and 5D, we showed that LHX2 loss led to elevated EPC percentages, confirming its function as an EPC-lineage suppressor. The serum-free conditions essential for EPC differentiation are incompatible with NSC survival, limiting investigation of LHX2's potential NSC regulatory functions in this experimental paradigm.

line 333: it is mentioned that Foxa2 depletion did not affect centriole amplification in early stages of EPC lineage differentiation but affected cilium growth, resembling Foxj1 deficiency. In figure 5H only cilia are shown and it's not clear what happens with Foxj1 regarding centriole amplification -it would be nice to have a clear comment and the comparison between the two molecules.

We have added the immunofluorescence image showing that *Foxj1* deficiency impaired centriole amplification into Figure 5J. A clear comment regarding the comparison between *Foxa2* and *Foxj1* have been made in the Results section (page 17, line 359-363) as follows:

While *Foxj1* depletion impaired centriole amplification in the early stages of EPC-lineage differentiation (**Figure 5J** and **Supplementary Figure 5E**) and the growth of cilium axoneme

in the later stages (**Figure 5K**), the loss of *Foxn4* specifically hampered cilium growth (**Figure 5H, J** and **K**).

line 366: Hes1 decrease is not obvious in the fig.6G. Is it statistically important?

Indeed, the change of *Hes1* expression was small, but it was statistically significant (Student's t-test, $P < 0.05$), indicating the effect was not random. We have added asterisk symbols indicating statistical significance in Figure 6G.

line 451: just a typo of in deed - the space should be deleted

Thanks. We have corrected the typo (page 23, line 495).

line 1237: the word facilitate gives a very strong statement- maybe just mention that play role in EPC lineage differentiation

We have changed 'facilitate' to 'play roles in' as suggested (page 67, line 1403).

line 1238: why different methodologies were used for McIdas and Lhx2?

While immunoblot analysis represents the gold standard for examining RNA interference efficacy due to its direct quantification of functional gene products (proteins), the absence of commercially validated McIdas antibody precluded this approach for *Mcidas*. As a compromise, we performed qPCR to assess *Mcidas* knockdown efficiency and immunoblot to examine *Lhx2* knockdown efficiency.

line 1240: promoted and inhibited are mentioned in reverse order according to your previous results

Sorry for the mistake. We have corrected this in the revised manuscript (page 67, line 1409).

line 1256: the time points that the experiment was performed should be included

Thanks for the suggestion. To improve the readability and the clarity, we have included the experimental scheme of RNA interference in Figure 5A.

line 1258, 1272 and 1280: add the b-catenin marker used as cell border marker

We have added this in the revised manuscript (page 68-69, line 1434, 1451-1452 and 1461).

line 1259 and 1282: identify the way you quantified the ECs- probably through g-tub accumulation?

We quantified the EPCs through centriole (gamma-tubulin) accumulation or multicilia (ARL13B) formation. Cells possessing more than four centrioles or multiple cilia were

classified as EPCs. The information has been included in the Methods section (page 39, line 821-822).

line 1269: Activated TFEB _delete the word of

We have deleted the word 'of' (page 69, line 1447).

line 1275: quantifications of Fig.7C should be added.

We have added the quantification to Figure 7C.

line 1285: Fig. 7G should be explained further, including more details for the experiment

The information on cell types, plasmid transfection, and sample amount for immunoblotting have been included in the Figure legend (page 69-70, line 1465-1469). More details for the immunoprecipitation experiment were provided in the Methods section (page 43, line 913-924).

line 1289: identify the details for the experiment (in which time points and the conditions that the siRNA was performed)

We have added the details for the RNA interference experiment (page 70, line 1471-1473).

line 1295: Maybe the statement that they are neuron-like cells should be done later when you use markers that show that.

We have changed 'neuron-like cells' to 'cells exhibiting neuron-like morphology' as suggested (page 70, line 1481).

line 1299 and 1302: Why different conditions are used in the two experiments (5 days serum starvation/ presence of serum)?

Utilizing the serum starvation-induced lineage-commitment protocol to generate EPCs (Figure 4F), we observed that overexpression of GFP-TFEB or GFP-TFEB (S210A) from day 0 to day +5 reduced the EPC ratio but promoted the percentage of cells exhibiting neuron-like morphologies (Figure 8B and B'), suggesting that TFEB overexpression induces an NSC-neuron fate. As most of neurons couldn't survive in DMEM without FBS or other essential nutrient supplement, we modified the protocol by changing the starvation medium (DMEM without FBS) to neuron-supportive medium (DMEM + 10% FBS) from day 0 to day +5 to prevent non-specific cell death in emerging neuronal populations (Figure 8C and C').

Fig. 1: for clustering in C some genes are shown in D. Based on which genes the clustering in E was done? A list should be provided.

We used the 'FindClusters' function in the Seurat package for clustering in Figure 1B and E (Figure 1D and G in the revised manuscript). Top 2,000 highly variable genes were selected

by the function for cell clustering. The gene list is provided in Supplementary Table 5. Cluster identities were determined through integrated analysis based on marker gene expression profiles shown in Figure 1D (Figure 1F in the revised manuscript) and the relative positions of each cluster on the bifurcating trajectory.

Fig.2: in line 29 you mention that bGPGs can give rise to nNSCs-NBs and nEPCs. So maybe in the illustration 2B bGPGs should have a neutral colour or some EPCs should be yellow in order to show that they derive from bGPGs.

To better illustrate the bGPC differentiation into nNSCs and nEPCs, we have changed the colors of nNSC and nEPC to gradient colors, which start from yellow.

Fig.8: the markers should be appeared in all images in order to be easier to follow and maybe the order of figures should be changed and show they graphical abstract of your results as a final point. Are there any qualifications for these experiments in order to do show how strong your result is?

We have added marker names to all images and placed the graphical abstract as a final point. The qualifications for these experiments were displayed in Figure 8A'-C' in the current version of the manuscript.

line 473: the term primary niche EPCs is not clear - do you mean neonatal as mentioned in the text above?

As we discussed the developmental origins of NSCs and EPCs in the adulthood here, the primary niche means mature EPCs. We have modified this to improve the clarity (page 26, line 542).

Line 511: you mention that you identified transcriptional factors that play it all in fate determination towards EPCs or NSC-NBS and refer to figure 4C that contains data only for EPCs.

Sorry for making the mistake. We have deleted 'or NSC-NBs' in the revised manuscript (page 27, line 577).

Line 529: you mention that Tfeb was upregulated both in vivo and in vitro, refering to figure 6A and B- could you please identify the experiments that were done in vivo?

As the differentiation pseudotime presented in Figure 6A and the differentiation trajectory displayed in Supplementary Figure 6A were derived from single-cell transcriptomic profiling of RGCs isolated from neonatal brains, we had classified them *in vivo* data. While these cells were of *in vivo* origin, the trajectory inference itself constitutes an algorithm-based computational reconstruction rather than direct experimental validation of *in vivo* differentiation processes. To prevent causing confusion, we have removed "both in vivo and in vitro" in the revised manuscript (page 28, line 595).

Line 572: I would suggest making also a hypothesis for possible treatments of hydrocephalus using this knowledge as you have stated in the abstract

We have discussed possible hydrocephalus treatment by targeting TFEB in the revised manuscript (page 30-31, line 636-652).

Line 585: add in the title that this is the staining protocol for FACS

We have modified the title to 'CD133-positive RGC isolation by FACS' (page 32, line 666).

Line 593, 658: you mention that culture medium contains 20% FBS. Later on in the protocol for example in line 673 you use 10% FBS. Why is this different percentage of FBS used? Have you characterised the cell culture of EPCs? If yes which is the percentage of ependymal cells when culturing under the conditions you mentioned above?

The experimental scheme of EPC culture system is shown in Figure 4F in the revised manuscript. Culture medium containing 20% FBS (a higher concentration) was maintained from day -5 to -2 to achieve 30-40% RGC confluence in the flask within 72 hours, a critical window for preserving differentiation potential towards EPC lineages. Prolonged proliferation beyond this duration was observed to compromise subsequent lineage specification efficiency. At day -2, cells were transferred onto glass-bottom dishes at a density of 5×10^5 cells/cm^2 (approximately 90% confluency). Serum concentration was reduced to 10% FBS during days -2 to 0, implementing a gradual reduction strategy to balance mitotic activity with cellular preparation for serum starvation. At 100% confluency, cultures were maintained in serum-free medium from day 0 onward to initiate EPC-lineage specification. Approximately 44% of the cells fulfilled differentiation into multiciliated EPCs at day +5 (see Reviewer Figure 3 in the next page). More details on EPC culture can be found in the Methods section (page 35-36, line 730-757).

Line 697: you mention that RNAi Was done in culture medium containing 10% FBS or starvation medium. How did you decide to do the procedure in starvation medium while differentiation has already started? It would be ideal to clarify in each experiment at which time point the RNAi has been performed in the corresponding figure.

Sorry for causing the confusion. Experimental scheme for RNAi followed by centriole staining or cilium staining have been provided in Figure 5A. The differentiation of RGCs into multiciliated EPCs proceeds through two mechanistically distinct phases: centriole duplication and subsequent ciliogenesis. To temporally resolve these processes, samples were harvested at day +2 and day +5 for centriole staining and cilium staining, respectively. The extended procedural timeline required for ciliogenesis analysis (days +2 to +5), combined with the transient efficacy of siRNA silencing, necessitated a dual-transfection strategy at day -1 and day +2 to sustain target gene knockdown throughout the differentiation process.

Under standard serum-starvation conditions, while 35.5% of cells displayed centriole amplification by day +2 (Figure 7B, 0 mM trehalose treatment group), only 6.7% exhibited

mature multiciliation (Reviewer Figure 3). This indicates the retention of differentiation potential in the majority of day +2 cells, as further evidenced by the subsequent increase to 44.3% multiciliation by day +5 (Reviewer Figure 3).

The time points when siRNA transfection was performed for qPCR, immunoblotting and RNA-seq experiments have been included in the corresponding figure legends.

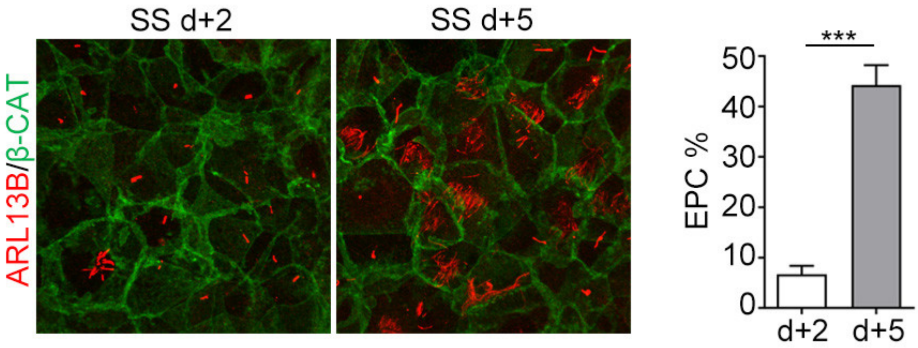

Reviewer Figure 3

References

1. Kyrousi C, Lygerou Z, Taraviras S. How a radial glial cell decides to become a multiciliated ependymal cell. Glia. 2017;65(7):1032-42. doi: 10.1002/glia.23118. PMID: 28168763.

2. Merkle FT, Tramontin AD, Garcia-Verdugo JM, Alvarez-Buylla A. Radial glia give rise to adult neural stem cells in the subventricular zone. Proc Natl Acad Sci U S A. 2004;101(50):17528-32. doi: 10.1073/pnas.0407893101. PMID: 15574494; PMCID: PMC536036.

3. Ortiz-Alvarez G, Daclin M, Shihavuddin A, Lansade P, Fortoul A, Faucourt M, et al. Adult Neural Stem Cells and Multiciliated Ependymal Cells Share a Common Lineage Regulated by the Geminin Family Members. Neuron. 2019;102(1):159-72 e7. doi: 10.1016/j.neuron.2019.01.051. PMID: 30824354; PMCID: PMC6449116.

4. Ramirez-Castillejo C, Sanchez-Sanchez F, Andreu-Agullo C, Ferron SR, Aroca-Aguilar JD, Sanchez P, et al. Pigment epithelium-derived factor is a niche signal for neural stem cell renewal. Nat Neurosci. 2006;9(3):331-9. doi: 10.1038/nn1657. PMID: 16491078.

5. Mirzadeh Z, Merkle FT, Soriano-Navarro M, Garcia-Verdugo JM, Alvarez-Buylla A. Neural stem cells confer unique pinwheel architecture to the ventricular surface in neurogenic regions of the adult brain. Cell Stem Cell. 2008;3(3):265-78. doi: 10.1016/j.stem.2008.07.004. PMID: 18786414; PMCID: PMC2613692.

6. Salvatierra J, Lee DA, Zibetti C, Duran-Moreno M, Yoo S, Newman EA, et al. The LIM homeodomain factor Lhx2 is required for hypothalamic tanycyte specification and differentiation. J Neurosci. 2014;34(50):16809-20. doi: 10.1523/JNEUROSCI.1711-14.2014. PMID: 25505333; PMCID: PMC4261103.

Reviewer #2:

This manuscript presented a single cell study of the developing ventricular zone (VZ) from mice and identified three distinct cellular states of radial glial cells. Several transcription factors were found to be essential for the EPC-lineage differentiation, in particular, TFEB was found to tune NSC/EPC bifurcation, independent of its canonical function as a master regulator of the lysosome biogenesis. These findings suggest the potential application of TFEB-targeted clinical drugs in VZ-related disorders, such as hydrocephalus and neurodegenerative diseases.

This is a comprehensive study of the molecular mechanisms relating to VZ with potentially interesting findings. Here are some comments for improvements.

1. Compromised study design and statistical analysis are major concerns in this study.

1) No biological replicates were presented for scRNAseq and bulk RNAseq. Also, only two replicates were used in ChIP-seq and plate-based scRNAseq. Power analysis is required to estimate the sample size.

We thank the reviewer for pointing out this problem. Our study is not really looking at effects upon certain treatments or under different conditions. Rather, it is about finding different cell types/subtypes and the trajectory along bGPC differentiation. Therefore, it is not possible for us to perform a power analysis in traditional sense. However, we do agree with the reviewer that more biological replicates are needed. In the revised manuscript now, we have incorporated biological replicates of P0 and P5 RGCs for scRNA-seq using 10X method, since it is a widely-used single-cell method in the community and generates more cells. While the two new replicates exhibited relatively reduced detected genes (Supplementary Figure 1B), pseudotime analysis via Monocle2 independently reconstructed conserved developmental bifurcations (Reviewer Figure 4), implicating the robustness of our data. Computational harmonization of technical variability using Harmony algorithm yielded an integrated dataset of 30,445 filtered cells exhibiting enhanced trajectory continuity (Figure 1E) compared to our initial submission. All downstream analyses were rigorously re-implemented based on the updated bifurcating trajectory and the conclusions remained unchanged.

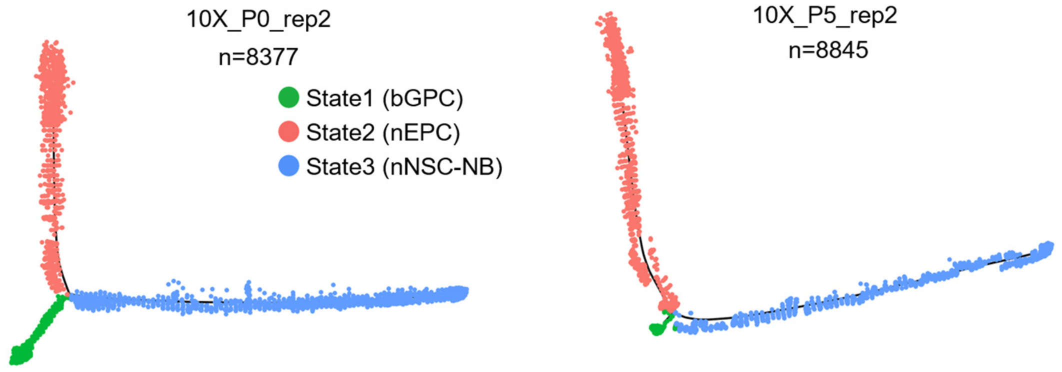

Reviewer Figure 4

We have added the third replicate for ChIP-seq and the UCSC genome browser tracks were presented in Figure 7H and Supplementary Figure 8E.

We are sorry for not being clear about the replicate information for bulk RNA-seq in the prior manuscript. We actually had three biological replicates each in the first place for *NC* or *Tfeb-si1* treated group for bulk RNA-seq. The information has been added to the Methods section (page 42, line 893-894).

All replicates were performed independently, and the final outcomes were similar. Therefore, we are confident that our results and conclusions are quite robust and reproducible.

2) No statistical analysis was presented in Methods although Student's t-test was described in Fig. 5-7 legends. What about other figures (e.g., Fig. 4F, Fig. 6B-C)? It is also unclear if all the experiment groups were compared to the NC group.

We thank the reviewer for pointing out this problem and sorry for the missing statistical information. We have included quantification results and statistical analysis for Figures 2F, 4G (Figure 4F in the original submission, with quantification now presented in Supplementary Figure 4F), 5C, 6B-C, 7A, 7C, 7D and Supplementary Figures 5E, 8A, 8C in the revised manuscript. All the siRNA-treated groups were compared to the NC group. We have added this information to the corresponding figure legends (page 68-69, line 1422-1423 and line 1443-1444).

3) No criteria (e.g., fold change and p value) were presented for the identification of differentially expressed genes in bulk RNAseq.

Differential expression analysis was performed using the DESeq2 package. The criteria for the identification of differentially expressed genes in bulk RNAseq were adjusted P value < 0.001 and $|\log2\text{fold change}| > 0.5$. We have included the information in the Methods section (page 42, line 894-896).

4) How were multiple testings corrected in RNAseq?

Multiple testings in RNAseq were corrected by calculating false discovery rate (FDR). The information has been added to the Methods section (page 42, line 896-897).

2. Why plate-based scRNAseq was used to confirm the findings of 10X scRNAseq? Is plate-based scRNAseq more reliable than 10X scRNAseq?

Sorry about the confusion. What we meant is that when we got the 10X scRNA-seq results, we would like to use independent experiments to validate the data. The plate-based method is cheaper for small-scale experiments with < 3000 cells compared to the commercial 10X Genomics platform. Therefore, we chose the plate-based method to perform independent experiments. More importantly, we would also like to avoid drawing conclusions that may be affected by potential doublets in the droplet system. The plate-based method used FACS as the single cell isolation method which is more accurate in terms of singlet identification compared to the droplet system. The final results are consistent between the two methods, so we are

confident about the results and conclusions.

3. Figure 5A: The labels are inconsistent between WB and bar plot. Please check if Mcidas instead of Lhx should be labelled in WB.

Sorry for causing the misunderstanding. In the old Figure 5A, the bar blot and the WB image were from different experiments: the bar blot showed the quantification of *Mcidas* expression obtained from qPCR experiment and the WB image showed *Lhx2* expression. In the current version of the manuscript, qPCR result of *Mcidas* expression was shown in Figure 5B, and the WB image demonstrating *Lhx2* expression with corresponding quantification was presented in Figure 5C.

4. What mouse strain was used in this study? The exact age of the neonatal mice should be provided for each experiment.

We thank the reviewer for pointing out this problem. The mouse strain used in this study is C57BL/6J. The strain and the age information have been included in the Methods section (line 655, 694, 697, 732, 829, 830, 844, 931).

5. Rescue experiments of applying the TFEB-targeted clinical drugs to animal models with VZ-related disorders such as hydrocephalus would enhance the conclusions of this study.

We thank the reviewer for the suggestion. As mouse model of hydrocephalus is not commercially available, we used the 5xFAD mouse, a well-established Alzheimer's disease model, for the rescue experiment. Rapamycin is a clinically approved mTOR inhibitor targeting TFEB activation. Consistently, treatment of RGCs with Rapamycin enhanced TFEB nuclear translocation in GPCs (Supplementary Figure 9A). We found that developmental-stage Rapamycin administration improved the spatial memory of the familial Alzheimer's disease model (Supplementary Figure 9E and F), showing the potential application of our study in treating VZ-related disorders. The text describing these results was included in the Results section (page 24-25, line 513-536).
