# Supplementary material for: Lineage trajectories and fate determinants of postnatal neural stem cells and ependymal cells in the developing ventricular zone
Source: PLoS Biol. 2025 Jul 30;23(7):e3003318. doi: 10.1371/journal.pbio.3003318 (PMC12327645; doi:10.1371/journal.pbio.3003318)
Supplement: Attachment — Submitted filename: Responses to Reviewers_250710.docx [file pbio.3003318.s020.docx]

**Responses to reviewers’ comments**

We thank both reviewers for their constructive comments. We provide below a point-by-point response to address the remaining concerns from the reviewers.

Black – reviewers’ comments

Blue – our responses to reviewers’ comments

Red – changes made to the prior version of manuscript

**Reviewer #1**:

Even though the modification in the revised version of the manuscript clarifies some of the issues raised, however there are several concerns that remain.

The manuscript offers limited insight into the lineage choice between adult NSCs and ependymal cells as many aspects have previously reported. Several studies have performed single cell experiments in the niche (eg Neuron. 2014 May 7;82(3):545-59; Cell, Volume 173, Issue 4, 1045 - 1057.e9q; Cell Reports, Volume 25, Issue 9, 2018, Pages 2457-2469.e8). In addition the common origin of adult NSCs and ependymal cells has recently been revealed by Spassky' and Buylla's lab as well as the role of Geminin GemC1 in this fate decision.

Both the *Cell* paper[1] and the *Cell Reports* paper[2] focused on cytoarchitecture of adult ventricular zone (VZ), employing scRNA-seq methods to unravel cellular heterogeneity. And the *Neuron* paper[3] utilized traditional microarray analysis rather than single-cell approaches to delineate transcriptomic differences between adult quiescent neural stem cells (qNSCs) and activated neural stem cells (aNSCs). Studies from Spassky' and Buylla's laboratories[4, 5] exploited lineage tracing techniques to clearly show that both adult NSCs and ependymal cells (EPCs) originate from radial glial cells (RGCs) in the VZ, thereby laying a foundation for our work.

We performed single-cell transcriptomic profiling of over 30,000 neonatal-stage RGCs from the VZ and uncovered a clear continuous bifurcating differentiation trajectory from glial progenitor cells (GPCs) to NSCs and EPCs **(Figures 1-3)**. Our analysis identified more than 200 transcription factors were specifically expressed or upregulated in the EPC branch **(Figure 4)**. Experimental validations revealed that NPAS1, FOXA2 and TFEB play roles in EPC-lineage differentiation by modulating multiciliogenesis **(Figures 5-8)**. To our knowledge, our manuscript represents the first single-cell transcriptomic atlas covering the developmental process of the VZ.

In addition in several cases the authors conclusions lead to misconceptions (eg line 117-118 the terminology GPCs is not used by the field and it is establish that EC mature slowly losing their radial glial cell characteristics, Ascl1 is not a NSC marker as it is expressed in TAPs).

We thank the reviewer for pointing this out. We have changed ‘GPCs’ to ‘RGCs’ in the revised manuscript (page 6-7, line 118-120). Transit amplifying progenitors (TAPs), also known as transit amplifying cells or neural progenitor cells, are derived from NSCs and subsequently differentiate into neuroblasts[3, 6, 7]. Together, NSCs, TAPs and neuroblasts form an NSC-lineage continuum. Previous studies showed that Ascl1, also known as Mash1, exhibits specific expression in both TAPs and NSCs[3, 6-8]. To make the description more precise, we have changed ‘NSC marker’ to ‘NSC-lineage marker’ (page 7, line 119-120).

Authors often are lead to conclusions without their experimental data to substantiate enough their conclusions (eg 166-167, 216-218, 191-194).

We have made the textual changes to temper the conclusions in these places (line 168-169, 193-196, 218-220).

McIdas does not suppress the conversion of GPCs into EPCs but does not allow to be fully differentiate (doi.org/10.1242/dev.172643).

We thank the reviewer for pointing this out. We have changed ‘depletion of *Mcidas* drastically suppressed the conversion of GPCs into EPCs’ to ‘depletion of *Mcidas* drastically suppressed multiciliogenesis of EPC precursors’ (page 16, line 340).

In addition Tfeb role is not clear as its expression is downstream of GemC1 key driver of EC commitment and differentiation and in the other hand depletion of Tfeb enhance EC differentiation (line 386). The authors do not provide solid data in order to claim "TFEB activation blocks GPCs specification in EPCs…" as they are experimental findings are based on a ex vivo Radial glial cell culture system.

Our findings demonstrated that the expression of TFEB is downstream of GemC1 (GMNC) (**Supplementary Figure 7C** and **D**) and increases as GPCs differentiate into EPCs (**Figure 6 A** and **B**). Considering that multiple motile cilia formation requires vast material consumption for their assembly and substantial energy expenditure for their beating, we proposed that TFEB serves as a molecular “brake” to prevent excessive multiciliogenesis of EPCs. This regulatory mechanism is crucial for EPCs to maintain their homeostasis due to the limited availability of cellular components and energy. The related discussion can be found in the manuscript (page 28, line 590-605).

We agree that our current data do not show that TFEB regulates GPC specification *in vivo*. We have added a sentence in the Discussion section to acknowledge the limitations of our study, which reads: ‘Although the mechanistic data is based on *ex vivo* RGC culture, these findings provide valuable preliminary insights into the poorly understood mechanisms governing multiciliogenesis’ (page 28-29, line 605-607).

**Reviewer #2:**

The authors have significantly improved the manuscript by addressing most concerns from this reviewer. However, the use of only two biological replicates in single cell RNAseq remains a concern. Typically, three or more biological replicates should be used. The authors should clearly claim the sample sizes for all experiments in figure legends.

We thank the reviewer for taking the effort to read our manuscript. We have now included the information on sample sizes for all experiments in the corresponding figure legends.

**References**

1.	Shah PT, Stratton JA, Stykel MG, Abbasi S, Sharma S, Mayr KA, et al. Single-Cell Transcriptomics and Fate Mapping of Ependymal Cells Reveals an Absence of Neural Stem Cell Function. Cell. 2018;173(4):1045-57 e9. doi: 10.1016/j.cell.2018.03.063. PMID: 29727663.

2.	Zywitza V, Misios A, Bunatyan L, Willnow TE, Rajewsky N. Single-Cell Transcriptomics Characterizes Cell Types in the Subventricular Zone and Uncovers Molecular Defects Impairing Adult Neurogenesis. Cell Rep. 2018;25(9):2457-69 e8. doi: 10.1016/j.celrep.2018.11.003. PMID: 30485812.

3.	Codega P, Silva-Vargas V, Paul A, Maldonado-Soto AR, Deleo AM, Pastrana E, et al. Prospective identification and purification of quiescent adult neural stem cells from their in vivo niche. Neuron. 2014;82(3):545-59. doi: 10.1016/j.neuron.2014.02.039. PMID: 24811379; PMCID: PMC4360885.

4.	Ortiz-Alvarez G, Daclin M, Shihavuddin A, Lansade P, Fortoul A, Faucourt M, et al. Adult Neural Stem Cells and Multiciliated Ependymal Cells Share a Common Lineage Regulated by the Geminin Family Members. Neuron. 2019;102(1):159-72 e7. doi: 10.1016/j.neuron.2019.01.051. PMID: 30824354; PMCID: PMC6449116.

5.	Redmond SA, Figueres-Onate M, Obernier K, Nascimento MA, Parraguez JI, Lopez-Mascaraque L, et al. Development of Ependymal and Postnatal Neural Stem Cells and Their Origin from a Common Embryonic Progenitor. Cell Rep. 2019;27(2):429-41 e3. doi: 10.1016/j.celrep.2019.01.088. PMID: 30970247; PMCID: PMC6499605.

6.	Pastrana E, Cheng LC, Doetsch F. Simultaneous prospective purification of adult subventricular zone neural stem cells and their progeny. Proc Natl Acad Sci U S A. 2009;106(15):6387-92. doi: 10.1073/pnas.0810407106. PMID: 19332781; PMCID: PMC2669396.

7.	Dulken BW, Leeman DS, Boutet SC, Hebestreit K, Brunet A. Single-Cell Transcriptomic Analysis Defines Heterogeneity and Transcriptional Dynamics in the Adult Neural Stem Cell Lineage. Cell Rep. 2017;18(3):777-90. doi: 10.1016/j.celrep.2016.12.060. PMID: 28099854; PMCID: PMC5269583.

8.	Beckervordersandforth R, Tripathi P, Ninkovic J, Bayam E, Lepier A, Stempfhuber B, et al. In vivo fate mapping and expression analysis reveals molecular hallmarks of prospectively isolated adult neural stem cells. Cell Stem Cell. 2010;7(6):744-58. doi: 10.1016/j.stem.2010.11.017. PMID: 21112568.
